# Designing high affinity target-binding peptides to HLA-E: a key membrane antigen of multiple myeloma

**DOI:** 10.18632/aging.103858

**Published:** 2020-10-28

**Authors:** Ying Yang, Mingli Sun, Zhaojin Yu, Jinwei Liu, Wei Yan, Zhuogang Liu, Minjie Wei, Hongtao Wang

**Affiliations:** 1 Department of Hematology, Shengjing Hospital of China Medical University, Shenyang, Liaoning, China; 2 Department of Pharmacology, School of Pharmacy, China Medical University, Shenyang, Liaoning, China; 3 Department of Pharmacy, Chifeng Municipal Hospital, Chifeng Inner Mongolia, China

**Keywords:** multiple myeloma, HLA-E, protein-protein interaction, key membrane antigen, high affinity target-binding peptides

## Abstract

Multiple myeloma (MM) is a plasma cell malignancy that is currently incurable. Finding new targets and designing drugs are crucial for the treatment of MM. The two datasets (GSE6691 and GSE39754) are used to screen highly expressed antigen on MM cells. HLA-E was an ideal target for it was a hub gene, and also located in one of the key clusters. Highly expression of HLA-E mRNA on MM cells was also confirmed by real-time qPCR testing the MM patients’ samples in Shengjing hospital. Crystal structure of HLA-E was obtained from Protein Data Bank (PDB ID: 3CDG) which was used to design targeting peptides with Molecular Operating Environment software. By analyzing interaction between CD94/NKG2A and HLA-E, a peptide with twelve amino acids was screened as a model peptide. Peptides library was constructed by randomly replaced non-key amino acid. Peptide-protein docking method was used to identify high affinity peptides. PEPTIDE 1-3 and model peptide were synthesized and identified the affinity to HLA-E by flow cytometer and confocal laser microscopy. At last, PEPTIDE3 (NALDEYCEDKNR) was found with the highest affinity. Taking all, HLA-E is a new treatment target, and PEPTIDE 3 is an ideal high affinity target-binding peptide candidate.

## INTRODUCTION

Multiple myeloma (MM) is a common plasma cell malignancy that accounts for more than 17% of hematological malignancies and 1.8% of all cancers in the United States [1]. Treatment of MM has been rapidly evolving, with not only new classes and generations of drugs (e.g., immunomodulatory drugs [IMiDs], proteasome inhibitors [PIs], monoclonal antibodies, histone deacetylase [HDAC] inhibitors) but also immunotherapy (e.g., CAR-T therapy) [2, 3]. However, MM remains an incurable disease, and patients suffer from relapse and refractory especially high-risk MM. So, finding new therapeutic targets and designing new drug candidates are crucial for the treatment of MM.

Microarray technology is widely used to investigate differential mRNA expression and identify biomarkers, which provides important clues to identify new treatment targets for many diseases, including MM [4, 5]. In the present study, we used bioinformatics tools to analyze the different expressed genes between MM cells and normal plasma cells to identify the new treatment target.

Targeting therapy has achieved great improvements in the treatment for malignancies, including MM (e.g. CD38 monoclonal antibody) [6]. At the meanwhile, peptide therapeutics have become an emerging method in the pharmaceutical industry aided by computational drug design [7, 8]. In our study, we are intending to design targeting affinity peptides to key membrane antigen in MM by computer software. And then evaluating the peptides’ affinity to the target by experiments. In future research, the targeting peptides will be linked to a chemical drug to produce a peptide-drug conjugate (PDC), which is a potential new drug candidate for the treatment of MM.

## RESULTS

### Identification of upregulated DEGs in MM

**Figure 1:**
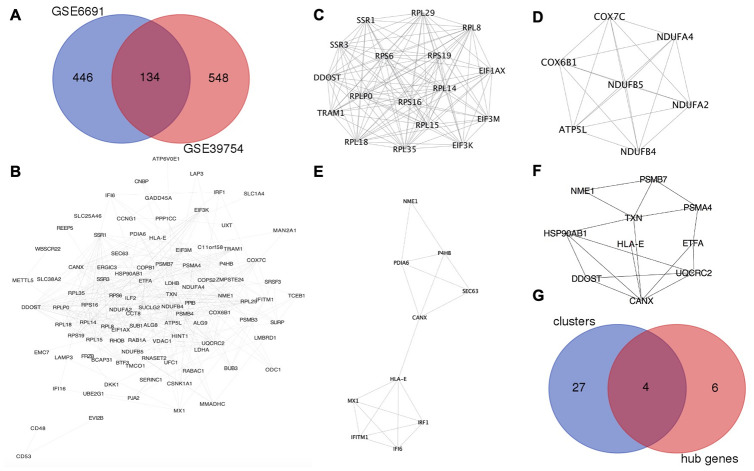
**HLA-E is a key gene in MM.** (**A**) Identification of upregulated DEGs in GSE6691 and GSE39754 datasets (Log_2_FC >1.5, *P* < 0.05). (**B**) The protein-protein interaction (PPI) network between co-upregulated DEGs in MM. (**C–E**). Modules in the PPI network with scores >4 and HLA-E in the cluster 3 (Figure 1E). (**F**). HLA-E is one of the hub genes in PPI network. (**G**) Overlapping genes between the key clusters and hub genes.

GSE6691 [9] and GSE39754 [10] datasets were found on Gene Expression Omnibus (GEO, http://www.ncbi.nlm.nih.gov/geo) database with MM patients and normal plasma cell samples. The number of upregulated DEGs on MM in the GSE6691 and GSE 39754 datasets were 580 and 682, respectively. The overlap between the two datasets consisted of 134 upregulated DEGs (Figure 1A).

### Identification of HLA-E as an anti-myeloma target by PPI network construction and hub gene analysis

The protein-protein interaction (PPI) network was constructed from the 134 co-upregulated DEGs though STRING (http://www.string-db.org/, version 10.5) [11] and visualized by Cytoscape (http://www.cytoscape.org/, version 3.6.0) (Figure 1B). Five modules were recognized as key clusters by molecular complex detection (MCODE, version 1.4.2) [12] clustering algorithm that comes with Cytoscape. Three of these modules had MCODE scores greater than 4 (Figure 1C–1E) and, thus, were considered key modules involved in the occurrence of MM. These three modules consisted of 31 genes. Hub genes were involved in the occurrence of myeloma, and identified by cytoHubba (version 0.1) [13] (Figure 1F). The four genes (DDOST, HLA-E, NME1, and CANX) were found in the overlap between the MCODE and cytoHubba analyses, which indicating that these genes may play an important role in MM (Table 1 and Figure 1G).

**Table 1:** List of hub genes in the key clusters.

**Antigens**	**Site**	**Structure of protein with interacted ligands**
NME1	Intracellular protein	NO
CANX	Membrane protein	NO
HLA-E	Membrane protein	PDB ID: 3CDG
DDOST	Membrane protein	NO

To design a peptide drug candidate for the treatment of MM, the suitable targets had to be membrane proteins. Thus, DDOST, HLA-E, and CANX were selected as potential targets. NME1 was excluded because it is an intracellular protein. However, a protein lacking available structural information could not be used as a target for the computational design of a peptide drug. Following a search of the RCSB Protein Data Bank (PDB, https://www.rcsb.org), HLA-E was the only key membrane protein suitable for designing anti-myeloma peptide drug-candidate. And *HLA-E* was in the cluster 3 and hub genes as shown in Figure 1E, 1F.

### Differential HLA-E expression in myeloma patients and controls

**Figure 2:**
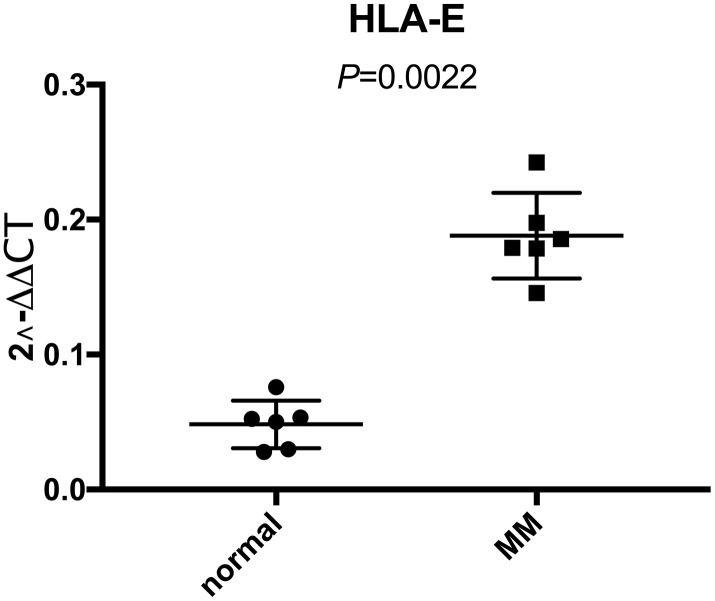
**Higher expression of HLA-E mRNA in CD138 positive myeloma cells than normal plasma cells detected with real-time qPCR.** The expression of HLA-E mRNA in normal plasma cells and MM cells were 0.048 ± 0.018 and 0.188 ± 0.032, respectively (*P* < 0.05).

We demonstrated that HLA-E was overexpressed on MM samples based on GEO database analysis. To further investigate the HLA-E expression on MM cells, we collected bone marrow samples (including 6 MM and 6 normal controls) in Shengjing hospital. After sorted by CD138 beats, HLA-E mRNA was detected. There were significantly higher HLA-E expression levels in the MM samples compared with the controls (*P* < 0.05, Figure 2).

### Design of HLA-E targeting peptides

**Figure 3:**
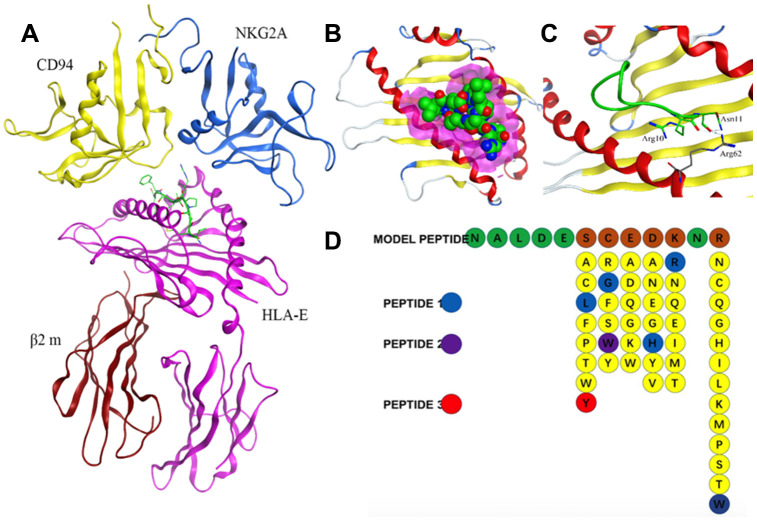
**Design of affinity peptides targeting HLA-E.** (**A**) Crystal structure of the HLA-E-CD94/NKG2A complex from PDB ID: 3CDG. (**B**) Interaction between model peptide and the key area of HLA-E (pink area represents the key interacting area). (**C**) Site view of the interaction between the model peptide and HLA-E; (**D**) Screening of a peptide library generated using the method of random replacing non-key site amino acids for high affinity peptides for HLA-E. The top three peptides (i.e., highest affinity and stability) were designated PEPTIDE 1, 2 and 3.

**Figure 4:**
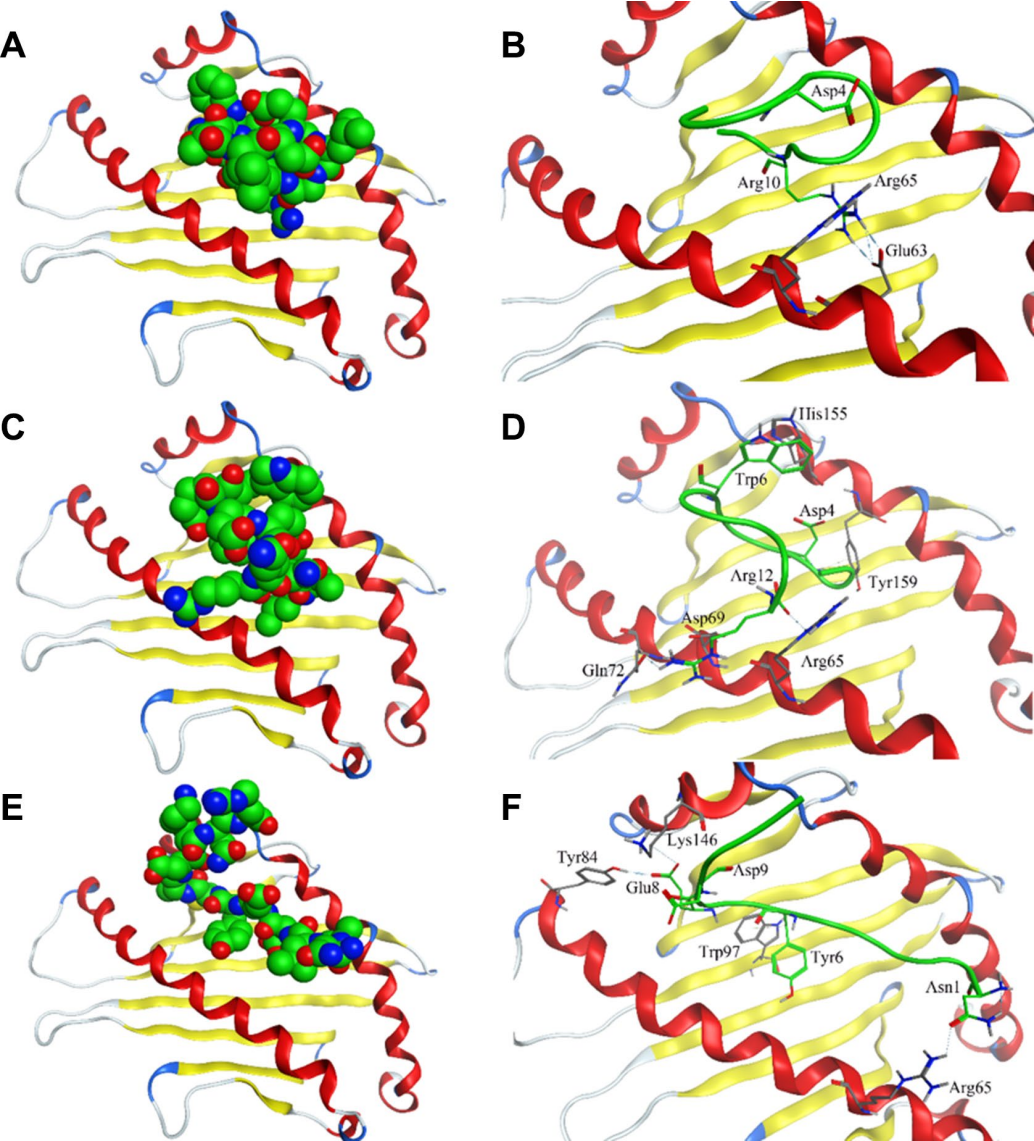
**Interaction of affinity peptides with HLA-E.** (**A, C, E**). The docking of PEPTIDE 1, 2 and 3 with the key area of HLA-E (PDB ID: 3CDG). (**B, D, F**). Site view of PEPTIDE 1, 2 and 3 docking with HLA-E. Dash lines, hydrogen bonds; Labeled residues, amino acids interacted between affinity peptides and HLA-E.

Crystal structure of HLA-E complex interacting with CD94/NKG2A (PDB ID: 3CDG) [14] had been used as the foundation for designing affinity target-binding peptides (Figure 3A) in the present study. The pocket in which HLA-E interacts with CD94/NKG2A was considered as the dominant site[15, 16]. The crystal structure data 3CDG was uploaded into the Molecular Operating Environment (MOE) software 2018.01 (Chemical Computing Group ULC, Montreal, Quebec, Canada) (http://www.chemcomp.com/). After refining and energy minimization steps, the area of the HLA-E complex that interacts with CD94/NKG2A was identified. Subsequently, a detailed analysis of the binding sites was carried out to investigate the favorable ligand-receptor contact regions. Both CD94 and NKG2A could interact with the pocket of HLA-E. The model peptides were obtained from CD94 or NKG2A which could interact with the pocket of HLA-E. Before docking, peptides conformation searching was done to make the peptide more realistic. Finally, a peptide segment containing 12 amino acids obtained from CD94 which was more stable, could be used as a model peptide for binding more accurately to the pocket of HLA-E. Protein docking showed that the model peptide could interact with the pocket of the HLA-E complex (Figure 3B; HLA-E pocket, pink surface; peptide, green, red, and blue balls). The site view of the model peptide docking with the pocket of HLA-E could ensure the interaction but with a low affinity for forming few chemical bonds (Figure 3C, Table 2). With the aim of enhancing the affinity of the peptides to HLA-E, a peptide library was constructed by randomly replacing non-key amino acids in the model peptide using MOE software (Figure 3D). Docked the top three peptides from peptide library (i.e., highest affinity and stability) with the pocket of HLA-E (Figure 4, Table 2). The results showed that PEPTIDE 3 was predicted to have the highest affinity and S value (Figure 4E, 4F and Table 2). PEPTIDE 1 and 2 also had higher affinity to HLA-E compared to the model peptide (Figure 4A–4D and Table 2). Based on the results, PEPTIDE 3 could markedly increase the bonding affinity, indicating that it was a potential peptide drug candidate for targeting HLA-E in myeloma.

**Table 2:** The amino acid sequence of affinity peptides and the interaction sites on HLA-E.

**Peptide**	**Sequence**	**S score**	**Interaction sites on HLA-E**
MODEL	NALDESCEDKNR	-36.7	H: R10-R62; N11-R62
PEPTIDE1	NALDELGEHRNW	-48.6	I: D4-R65; H: R10-E63
PEPTIDE2	NALDESWEDKNR	-47.4	H: R12-R65; R12-Q72; a: W6-H155; D4-Y159; i: R12-D69; R12-R65
PEPTIDE3	NALDEYCEDKNR	-55.9	H: N1-R65; E8-Y84; E8-K146; a: Y6-W97; i: D9-K146; D: E8-Y84; C7-F116

**Figure 5:**
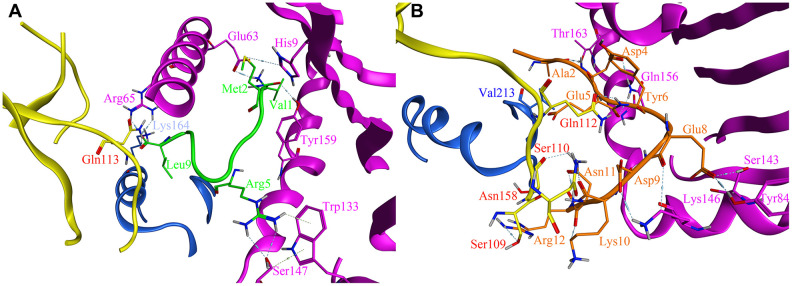
**The site view of the interactions between leader peptide (shown as green) and the designed PEPTIDE 3 (shown as orange) with the HLA-E proteins (HLA-E protein is shown as purple, CD94 protein is shown as yellow, NKG2A protein is shown as blue).** All hydrogen bonds and residues are labeled. (**A**) the site view of leader peptide docked with HLA-E; (**B**) the site view of PEPTIDE 3 with HLA-E.

As previous report, only with leader peptide could HLA-E protein express on the surface of the cells [16]. There were several ways HLA-E could obtain the leader peptide (nine amino acids with different sequences) [17]. We compared the affinity between the designed affinity peptide and leader peptide with HLA-E. The final best pose with the highest score was analyzed and compared to the binding mode of the PEPTIDE 3 with the HLA-E protein. According to the binding free energy of HLA-E-peptide complex, we found the designed PEPTIDE 3 was binding more stronger than the original leader peptide (PEPTIDE 3 with the highest score of 17.06 kcal/mol and leader peptide with the highest score of 15.26 kcal/mol). In addition, the interactions between peptides and HLA-E were analyzed. As seen in Figure 5, the designed PEPTIDE 3 (Figure 5B) had more interactions with HLA-E protein than the original peptide (Figure 5A). Figure 5A showed that leader peptide can form hydrogen bonds between Val1 and Tyr159, Met2 and Glu63, His9, Arg5 and Trp133, Ser147, Leu9 and Arg65 of HLA-E. Hydrogen bonds between Leu9 and Gln113 of CD94, Lys164 of NKG2A protein could also be formed to increase the binding energy. However, the designed PEPTIDE 3 could form more interactions which include hydrogen bonds between Ala2, Asp4, Glu5, Glu8, Asp9, Lys10, Asn11, Arg12 and Thr163, Gln156, Ser143, Tyr84, Lys146 of HLA-E protein. It could also form several hydrogen bonds between PEPTIDE 3 and CD94 protein, such as Glu5, Asn11, Arg12 and Gln112, Ser110, Asn158, Ser109 (As seen in Figure 5B). The computational study indicated that the designed PEPTIDE 3 bound more stronger to the HLA-E protein and had more binding free energy.

### Verify the targeting peptides’ affinity against HLA-E by FCM and confocal laser microscopy

**Figure 6:**
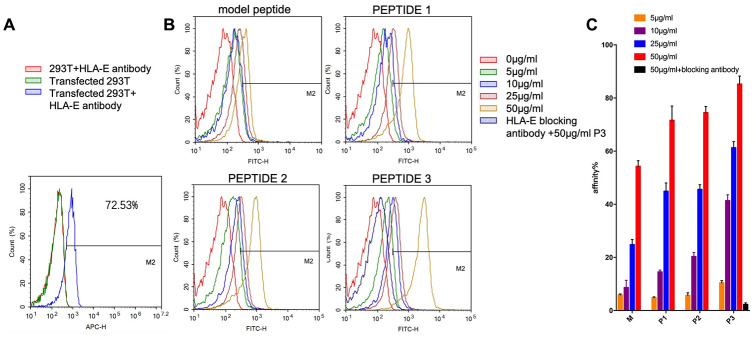
**The affinity of targeting peptides increased with the growing of concentration and could be blocked by HLA-E blocking antibody.** (**A**) 293T cells did not express HLA-E, and plasmid transfect 293T cells expressed HLA-E (*p*<0.01). (**B**) the affinity of model peptide and PEPTIDE 1-3 increased with the increasing of concentration. Precultured transfected 293T cells with HLA-E blocking antibody, the affinity of P3 disappeared. (**C**) The statistical chart about the affinity of peptides to HLA-E. All repeated for three times.

The expression of HLA-E on the surface of 293T cells and HLA-E plasmid transfected 293T cells were detected by flow cytometer (FCM, ACEA NovoCyte^TM^, Hangzhou, China). The result showed that 293T cell line didn’t express HLA-E protein on cell surface (Figure 6A). The expression rate of HLA-E on 293T cell line was 72.53% after HLA-E plasmid transfection (Figure 6A). The HLA-E high expressed 293T cells and 293T cells were incubated with different concentration fluorescein isothiocyanate (FITC) labeled model peptide and PEPTIDE 1-3 for 1h. And then detected the affinity of targeting peptides to HLA-E by FCM. The result showed that with the concentration growing, more peptide could be detected in HLA-E high expressed 293T cells (Figure 6B–6E). PEPTIDE 3 had the highest affinity to HLA-E which was consistent with computational result (Figure 6E, 6F). After that HLA-E blocking antibody was used to incubate with HLA-E plasmid transfected 293T cells for 30mins, and then incubated with 50μg/ml PEPTIDE 3. The result showed that fluorescence intensity nearly disappeared (Figure 6E, 6F).

**Figure 7:**
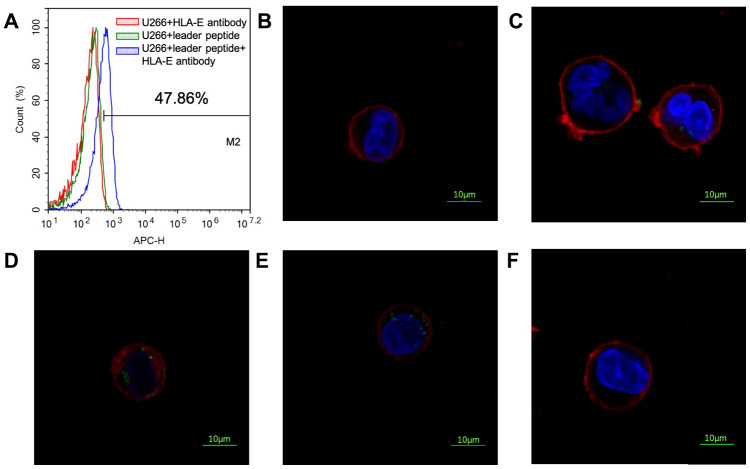
**Affinity of PEPTIDE 3 to HLA-E high expressed U266 cells.** (**A**) HLA-E protein was not detected on U266 cells; pretreated U266 cells with leader peptide could induce HLA-E expression. (**B**) 5μg/ml PEPTIDE 3 interacted with HLA-E high expressed U266 cells; (**C**) 10μg/ml PEPTIDE 3 interacted with HLA-E high expressed U266 cells; (**D**) 25μg/ml PEPTIDE 3 interacted with HLA-E high expressed U266 cells; (**E**) 50μg/ml PEPTIDE 3 interacted with HLA-E high expressed U266 cells obviously; (**F**) 50μg/ml PEPTIDE 3 could not interacted with HLA-E blocking antibody pre-treated HLA-E high expressed U266 cells. The figures were representing ones and all repeated for three times.

U266 cell line is a common myeloma cell line. HLA-E protein could not be detected on the surface of U266 cell line by FCM (Figure 7A). As previous experiment introduced that HLA-E protein need leader peptide to express on myeloma cells surface [18]. With this protocol, HLA-E protein was detected on the surface of U266 cells by pretreating with leader peptide (Figure 7A). This HLA-E expressed U266 cell line was used to detect the FITC labeled peptides’ affinity by confocal laser microscope. PEPTIDE 3 which had highest affinity to HLA-E was used in this part. With the concentration growing, more FITC labeled PEPTIDE 3 could be detected on the HLA-E expressed U266 cells (Figure 7B–7E). When HLA-E expressed U266 cells pre-treated with HLA-E blocking antibody, the affinity of P3 to HLA-E disappeared (Figure 7F).

## DISCUSSION

The treatment of MM has achieved great progress with the use of new drugs, even for some of the refractory and relapse patients [19]. With the improvements in biological knowledge, therapeutic options are able to overcome the high-risk behavior that represents an unmet clinical need [20]. Novel therapeutic way, targeting the neoplastic clone and improving its immune microenvironment are important for the treatment of MM [21, 22]. In order to find new treatment target, identifying high expressed antigen on the surface of MM cells is important. GEO datasets provide mRNA expression data for many types of diseases and normal tissues. This database makes it possible for us to increase the number of samples so that we could evaluate to discover a new therapeutic target for MM. With bioinformatics analysis, we identified four candidate therapeutic targets (DDOST, HLA-E, NME1, and CANX) for the treatment of MM. One of the most effective techniques to achieve the selective delivery of drugs is based on targeting overexpressed receptors on the surface of cancer cells. NME1 was eliminated for it was not a membrane protein, and thus would have required the ability of drugs to penetrate the membrane to be effective. Because our approach relied on rational computational design of the peptide drug, structural information for the candidate targets was required. Thus, DDOST and CANX were eliminated for had no structural information with ligands. As a result, HLA-E was the only candidate drug target in the present research. The high expression of HLA-E mRNA was identified in bone marrow samples from MM patients in Shengjing Hospital. Thus, it represents a potential new target for the treatment of myeloma.

HLA-E is characterized by lower polymorphism [23]. and plays a critical role in the immune response by both inhibiting and activating the function of natural killer (NK) cells [24]. The effects of HLA-E on NK cells are mediated through its binding with either CD94/NKG2A (an inhibitory receptor) or NKG2C (an activating receptor). The affinity of HLA-E to CD94/NKG2A is much greater than CD94/NKG2C [25, 26]. So, HLA-E highly express on the surface of cells can inhibit the killing function of NK cells [27]. Thus, high HLA-E expression levels on the surface of tumor cells could inhibit the function of NK cells, which might be an important mechanism underlying tumor escape. Moreover, several malignancies and viral diseases express high levels of HLA-E, including hematological diseases [28–32]. In recent years, immunotherapy has been used to treat many kinds of malignancies successfully, even relapse patients [33]. Taken together, HLA-E is not only a target to find MM cells but also an immune checkpoint. Targeting HLA-E may could restore and enhance the immune function to give a new way for treating many malignant diseases including MM.

Therapeutic peptides have gained a wide range of applications in medicine because of their safety, tolerability, high selectivity, and good efficacy [34]. Computational techniques can identify the new peptide drugs against specific targets and evaluate the bonding affinity before they are synthesized in laboratory [35, 36].

In the present study, we designed HLA-E targeting affinity peptide drug by analyzing the interaction between HLA-E and CD94/NKG2A. Previous works showed that HLA-E had very limited allelic polymorphism [23]. For designing HLA-E targeting peptide, low polymorphism was more suitable. HLA-E usually presented peptides derived from the leader sequence of other HLA class I molecules. However, it became evident that HLA-E ligands were not restricted to the leader peptides of HLA class I molecules [37–40]. 3CDG from PDB website provided structure of the interaction between HLA-E and its ligand with high resolution. Peptides obtained from CD94, NKG2A and leader peptide were docking with key area of HLA-E respectively. At last, a peptide with 12 amino acids was found forming the most bonds with HLA-E and considered as a model peptide. With the strategy of randomly replacing non-key amino acids to enhance the affinity of peptides [41]. A peptide library was built. And we evaluated the peptides’ affinity to the target by a protein docking program. The top three peptides were subjected to molecular docking analysis, and PEPTIDE 3 (NALDEYCEDKNR) was found to have the highest affinity for HLA-E. There were several studies highlighted the critical importance of peptides, most derived from the leader sequence of HLA-G, emphasizing the role of HLA-E and CD94/NKG2A system [42, 43]. To our surprise, PEPTIDE 3 was even binding stronger to HLA-E than original leader peptide for forming more interaction with HLA-E protein. If PEPTIDE 3 had stronger affinity to HLA-E, it might interfere the steady expression of HLA-E on the surface of MM cells. At that time, NK cells could recognize MM cells and kill it. The immune function of NK cells will recover. Up to now, high affinity peptides in theory were identified.

In order to verify the affinity of peptides to HLA-E. The potential high affinity peptides (model peptide and PEPTIDE 1-3) were synthesized and labeled by FITC fluorescein. We built HLA-E high expressed cell lines 293T and U266 by plasmid transfection and leader peptide pretreating respectively. FCM and confocal laser microscopy were used to detect the FITC labeled HLA-E targeting peptides. The result verified the computer work, PEPTIDE 3 had the highest affinity to HLA-E. Indicating PEPTIDE 3 could be considered as a potential targeting peptide drug candidate to target MM cells.

However, there are still some limitations in our study. We verified HLA-E high expression on some MM patients, while the number of patients was not large enough. Moreover, the prognostic significance of HLA-E on MM patients was not discussed. We also need to clarify the side effect of PEPTIDE 3, especially off-target effect in vivo experiment. In further, we will do the biological effects of PEPTIDE 3 not only on MM cells cell lines and in myeloma primary cells but also in vivo.

In summary, the present study has identified HLA-E as a new therapeutic target for MM and designed affinity peptides targeting it. PEPTIDE 3 has the strongest affinity to HLA-E. In future studies, the affinity peptide could also be used to produce peptide-drug-conjugates (PDC) for the target treatment of MM. The present work provides a new way for the treatment of MM.

## MATERIALS AND METHODS

### Microarray data

GSE6691 and GSE39754 datasets were downloaded from the GEO database and combined for analysis. The datasets included bone marrow samples from 182 MM patients and 11 healthy volunteers (control samples).

### Identification of upregulated differentially expressed genes (DEGs)

The upregulated DEGs in MM samples compared to the controls were screened using GEO2R (http://www.ncbi.nlm.nih.gov/geo/geo2r). Log2FC (fold change) > 1.5 and P-value <0.05 were set as standard criteria. The co-DEGs between GSE6691 and GSE39754 were identified by Venn diagram online (http://bioinformatics.psb.ugent.be/webtools/Venn/).

### PPI network construction and cluster analysis

PPI network of co-upregulated DEGs was constructed using the STRING online database. A combined score greater than 0.4 was considered statistically significant. Cytoscape was used to visualize the PPI networks. The whole PPI network was clustered into several key modules using MCODE. The criteria for selection were as follows: MCODE score > 4, degree cut-off = 2, node score cut-off = 0.2, k-core = 2, and maximum depth = 100.

### Identification of hub genes

CytoHubba was employed to determine the hub genes of the PPI network. In this study, BottleNeck calculations were used to identify the top ten hub genes.

### Patients and bone marrow samples

Bone marrow samples from six MM patients and six non-hematological malignant patients were collected from the Hematological Department, Shengjing Hospital of China Medical University from Sep 2016 to Jun 2017 with informed consent. All experimental protocols were approved by the Ethics Committee in Shengjing Hospital. MM cells were purified with CD138 magnetic beads using positive selection according to the manufacturer’s instructions (Miltenyi Biotec, Germany) [44].

### Real-time quantitative RT-PCR (RT-qPCR)

Total RNA was extracted using Trizol (TaKaRa, Japan). Reverse transcription (2.5 μg total RNA) was performed using a cDNA synthesis kit (TaKaRa, Japan). qRT-PCR was performed with SYBR Premix EX Taq (TaKaRa, Japan) using the QuantStudio 3 real-time PCR system (ThermoFisher, USA). RT-qPCR primers were synthesized as described [45]: HLA-E forward primer, 5'-ccgtcaccctgagatgga-3'; HLA-E reverse primer, 5'-agcaatgatgcccacgat-3'. β-actin was used as a reference and amplified using primers 5'-ccaaccgcgagaagatga-3' and 5'-ccagaggcgtacagggatag-3'. qRT-PCR was performed by denaturation at 95°C for 3 min followed by 40 cycles of denaturation at 95°C for 12 sec and annealing at 62°C for 40 sec. The relative levels of HLA-E expression were calculated as ΔCt = Ct(gene) - Ct(reference). The 2^-ΔΔCt^ method was used to calculate the fold-change of gene expression.

### Design of high-affinity targeting peptides against HLA-E

Crystal structure of homo sapiens’ HLA-E complex interacting with CD94/NKG2A was obtained from the PDB website. The result showed that there were two crystal structures available listed as PDB code 3CDG and 3CII. 3CDG with the higher resolution which would be used as the foundation for the design of affinity targeting peptides. Interactions between proteins and peptide-protein docking were performed by MOE software. To design affinity peptides targeting HLA-E, we firstly fulfilled the structure preparation of the HLA-E-CD94/NKG2A complex by addition of hydrogen, removal of H_2_O molecules, and energy minimization. Next, we identified the key interacting area between HLA-E and CD94/NKG2A by analyzing the bonding and mining literature data. A peptide obtained from CD94/NKG2A was used as a model peptide for interaction with the HLA-E pocket. The peptide library was constructed based on the model peptide using the residue scan module in MOE software for random replacement of non-key amino acids. All peptides with predicted high affinity for HLA-E were optimized as follow. Firstly, conformational searching of these peptides was conducted with LowModeMD method; Secondly, docking was conducted based on two steps (placement and refinement) of Dock module in MOE. Triangle matcher and rigid receptor method were used in these two steps. 100 poses would produce in the placement step and at last exported 10 poses. At last, London dG and GBVI/WSA dG scoring function were used to estimate the affinity of peptide to HLA-E. The binding affinity of these peptides could be predicted by forming interaction bonds and binding free energy (S value obtained from docking simulations).

### Verify the targeting peptides’ affinity against HLA-E by FCM

293T cells as a tool cell line which collected by our laboratory were used for construction HLA-E high express cell lines by transfecting HLA-E plasmid with Lipofectamine 3000 (Invitrogen) as previous report [46]. Affinity peptides which labeled by FITC were purchased from Chinese Peptide (Hangzhou, China). The expression of HLA-E was detected using antihuman fluorescent monoclonal antibody allophycocyanin (APC) and performed by flow cytometer (FCM, ACEA NovoCyte^TM^, Hangzhou, China). The HLA-E antibodies were purchased from Biolegend (clone: 3D12, San Diego, CA, USA). 5μg/ml, 10μg/ml, 25μg/ml, 50μg/ml model and PEPTIDE 1-3 were cultured with HLA-E high expressed 293T cells and 293T cells. Then affinity of peptides against HLA-E were detected by FCM. Firstly, a ‘live gate’ was circled. Secondly, Cells were first gated by their FSC/SSC properties, and then the percentage of each concentration FITC-labeled peptides were computed (Supplementary Figure 1).

### Verify the targeting peptides’ affinity against HLA-E by confocal laser microscopy

U266 cells was a common kind of myeloma cell lines which could be inducted to express HLA-E by adding leader peptide as previous report [28]. U266 cells were incubated with 500 μM of HLA-B7 (VMAPRTVLL) or a control peptide (VGRGRAFVLI) for 12 h at 37 °C. Cultured U266 cells without peptide or with the solvent of the peptides (DMSO) considered as negative control. HLA-E expression was determined by FCM.

HLA-E high expressed U266 cells and U266 cells were used for verification the affinity of the targeting peptides against HLA-E by confocal laser microscopy [47]. Preparations for microscopic observations as follows: FITC-labeled targeting peptides solution of 1μL (50mg/ml) and 1mL cells (4×10^5^) were mixed into a six-hole plate for 1h and then fixed by polyformaldehyde for 30mins. Membrane and nuclei were dyed by DLI and DAPI for 30mins after washing and resuspending the cells with phosphate buffer solution (PBS). Nikon C2 confocal microscope was used for microscopic observations [48]. The sample was stored in dark place at 4°C, and then observation was carried at 20mins after dyeing.

### Statistical analysis

The data were expressed as the mean ± SD and two group statistical comparisons of means were calculated using the Student's *t*-test (SPSS software 23.0). A P-value < 0.05 was considered statistically significant.

## Supplementary Material

Supplementary Figure 1
