# Supplementary material for: Designing high affinity target-binding peptides to HLA-E: a key membrane antigen of multiple myeloma
Source: Aging (Albany NY). 2020 Oct 28;12(20):20457–70. doi: 10.18632/aging.103858 (PMC7655190; doi:10.18632/aging.103858)
Supplement: Supplementary Figure 1 [file aging-12-103858-s001..pdf]

SUPPLEMENTARY FIGURE

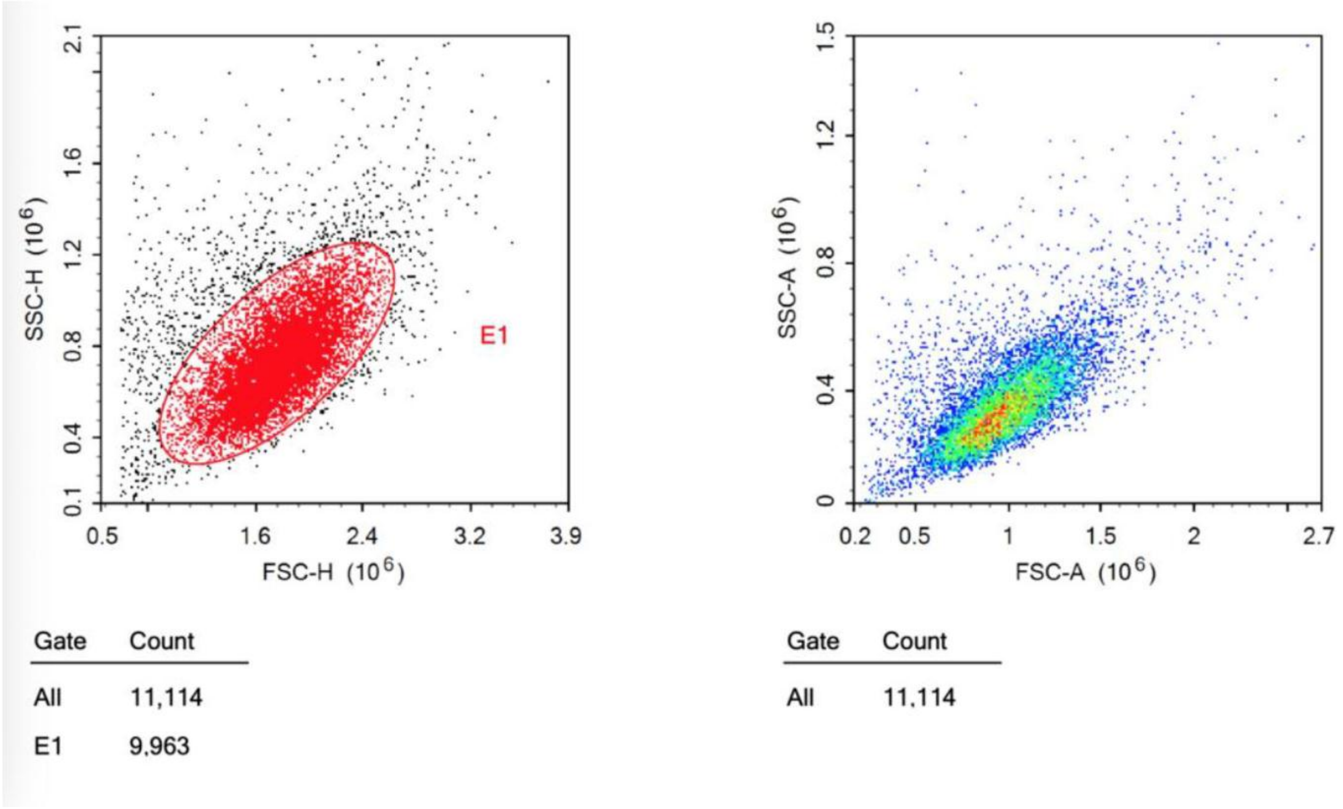

Supplementary Figure 1. The way to circle cells. First, a 'live gate' was circled. Cells were first gated by their FSC/SSC properties and then, the percentage of each concentration FITC-labeled peptides were computed.
